# A human forebrain organoid model phenocopies dysregulated RNA and protein homeostasis in ALS/FTD-associated TDP-43 proteinopathies

**DOI:** 10.1101/2025.11.09.687455

**Published:** 2025-11-10

**Authors:** Qi Zhang, Meng Liu, Xiaojuan Fan, Natalie Chin, Yue Xu, Jessica Suh, Soukaina Amniouel, Kaari Linask, Jizhong Zou, Markus Hafner, Lichun Ma, Wei Zheng, Yihong Ye

**Affiliations:** Therapeutic Development Branch, National Center for Advancing Translational Sciences, National Institutes of Health, Bethesda, MD, 20892, USA; Cancer Data Science laboratory, National Cancer Institute, National Institutes of Health, Bethesda, MD, 20892, USA; RNA Molecular Biology Laboratory, National Institute of Arthritis and Musculoskeletal and Skin Disease, National Institutes of Health, Bethesda, MD, 20892, USA; Laboratory of Molecular Biology, National Institute of Diabetes, Digestive, and Kidney Diseases, National Institutes of Health, Bethesda, MD, 20892, USA; Laboratory of Molecular Biology, National Institute of Diabetes, Digestive, and Kidney Diseases, National Institutes of Health, Bethesda, MD, 20892, USA; Therapeutic Development Branch, National Center for Advancing Translational Sciences, National Institutes of Health, Bethesda, MD, 20892, USA; Therapeutic Development Branch, National Center for Advancing Translational Sciences, National Institutes of Health, Bethesda, MD, 20892, USA; iPSC Core, National Heart, Lung, and Blood Institute, National Institutes of Health, Bethesda, MD, 20892, USA; iPSC Core, National Heart, Lung, and Blood Institute, National Institutes of Health, Bethesda, MD, 20892, USA; RNA Molecular Biology Laboratory, National Institute of Arthritis and Musculoskeletal and Skin Disease, National Institutes of Health, Bethesda, MD, 20892, USA; Cancer Data Science laboratory, National Cancer Institute, National Institutes of Health, Bethesda, MD, 20892, USA; Therapeutic Development Branch, National Center for Advancing Translational Sciences, National Institutes of Health, Bethesda, MD, 20892, USA; Laboratory of Molecular Biology, National Institute of Diabetes, Digestive, and Kidney Diseases, National Institutes of Health, Bethesda, MD, 20892, USA

**Keywords:** iPSC-derived forebrain organoid, TDP-43 proteinopathies

## Abstract

**Background:**

TAR DNA-binding protein 43 (TDP-43) proteinopathy is a central hallmark of amyotrophic lateral sclerosis (ALS) and frontotemporal dementia (FTD), yet current experimental models fail to reproduce the full pathological spectrum without external stress or TDP-43 overexpression. This study aims to establish a human induced pluripotent stem cells (iPSC)-derived system that spontaneously manifests TDP-43 pathology driven by an ALS-associated TDP-43 mutation.

**Methods:**

We generated forebrain 3-D organoid cultures from iPSC carrying the TDP-43 K181E patient mutation. Single-cell RNA sequencing was used to define transcriptional alterations across cell types, and enhanced crosslinking immunoprecipitation (eCLIP) was applied to examine the global RNA binding and splicing defects in mutant organoids. We further used immunostaining, RT-PCR and biochemical assays to confirm TDP-43 proteinopathy and validate findings from the multi-omics analyses.

**Results:**

The TDP-43 K181E organoids recapitulated key disease features, including cytoplasmic p-TDP-43 accumulation, RNA dysregulation, and cryptic exon inclusion. Single-cell analysis revealed a population of immature neurons with enhanced neuroinflammation and altered translation capacity. Comparative transcriptomics showed that the ALS mutation-induced transcriptional changes strongly overlap with those in ALS patient-derived brains. eCLIP analysis showed that mutant TDP-43 exhibited altered RNA-binding specificity, resulting in widespread RNA mis-splicing and cryptic exon inclusion. RT-PCR confirmed *PRDM2*, a gene regulating cell senescence, is mis-spliced in mutant cells. These defects collectively disrupt neuronal homeostasis and cell-cell communications.

**Conclusions:**

Our iPSC-derived forebrain organoid model displays spontaneous TDP-43 proteinopathies and associated molecular dysfunctions without artificial manipulation. The model offers a robust platform for dissecting the mechanisms of TDP-43-mediated neurodegeneration and advancing therapeutic discovery in ALS and FTD.

## Introduction

The neurodegenerative disorders amyotrophic lateral sclerosis (ALS) and frontotemporal dementia (FTD) are united by the pathological mis-localization and aggregation of TAR DNA-binding protein 43 (TDP-43) in affected neurons and glial cells (1, 2). TDP-43 aggregation is present in approximately 97% of ALS and 45% of FTD cases, as well as in subsets of other neurodegenerative conditions including Alzheimer’s disease and chronic traumatic encephalopathy. These diseases are now collectively termed TDP-43 proteinopathies (1, 3). Despite extensive research, the cellular and molecular mechanisms underpinning TDP-43 aggregation and its contribution to neurodegeneration remain poorly understood. A major gap in the field is the lack of physiologically relevant disease model.

TDP-43 is a nuclear RNA-binding protein with essential roles in RNA metabolism, including RNA splicing, transport and stability (4, 5). It contains an N-terminal dimerization domain, two RNA-recognition motifs (RRM1 and RRM2), and a C-terminal low-complexity domain (LCD) (6). The LCD of TDP-43 is known to undergo liquid-liquid phase separation (LLPS) (6, 7); Under pathological or stress conditions, this property is altered such that TDP-43 undergoes a liquid-to-solid phase transition (7, 8). This leads to abnormal nuclear RNA regulation while generating cytoplasmic TDP-43 aggregates (4, 6, 9). Notably, existing experimental models often rely on artificial manipulations such as TDP-43 overexpression or stress induction (10–14). While these models have provided valuable insights into the potential disease mechanisms, they often fail to recapitulate endogenous TDP-43 aggregation and other disease-associated pathologies, therefore, are not ideal disease models for therapeutic evaluation or mechanistic study.

Recent technology advancement in induced pluripotent stem cells (iPSCs) and CRISPR-mediated gene editing, combined with the establishment of three-dimensional (3D) organoid models have provided new opportunities for disease modeling (15–17). These 3D cell assemblies, derived from CRISPR-engineered iPSC cells bearing endogenous disease mutations, can generate distinct cell types that self-assemble into brain-like structures with extensive cell-cell interactions. Thus, organoids often better mimic the cellular complexity and organization of the human brain.

To date, ~70 genetic mutations in *TARDBP* have been linked to ALS or FTD. While the majority of ALS/FTD-associated mutations are located within the LCD, many are located in or near the RRMs. These TDP-43 mutants are often defective in RNA-binding (6), which indirectly affect their biochemical properties in LLPS or liquid-solid regulation (18). Of particular interest is a recently identified catastrophic mutation (K181E) in an ALS/FTD family, which a 38-year-old man and 76-year-old father both developed major symptoms, and passed away within 80 months and 36 months respectively (19). The TDP-43 K181E mutant cannot bind the canonical TDP-43 binding sequence in RNA, yet it can still undergo LLPS in vitro (20, 21). In cells, overexpressed TDP-43 K181E mutant is hyperphosphorylated and forms large irregularly shaped aggregates without external stress (19). However, it is unclear how this mutation causes neuronal cell dysfunction.

In this study, we attempted to model TDP-43 K181E-associated neurodegeneration by introducing the TDP-43 K181E mutation into a well-characterized iPSC line (22, 23) since patient-derived stem cell lines were unavailable due to demise of both patients. We grew and differentiated these cells into neurons or 3-D brain-like organoids and found that 3-D organoids bearing the disease mutation display spontaneous TDP-43 phosphorylation and cytoplasmic aggregation. Our multi-omics-based approach further revealed key molecular signatures in these organoids closely resembling patient phenotypes including RNA dysregulation and neuroinflammation. Importantly, we identified previously unknown RNAs as TDP-43 substrate whose mis-splicing may contribute to TDP-43 proteinopathies. Our study established a novel forebrain model of TDP-43 proteinopathy for further dissecting the pathological mechanisms of ALS/FTD and developing effective therapeutics.

## Materials and methods

### Stem cell gene editing with knock-in K181E patient mutation

1.2×10^6^ Kolf2.1 NGN2 cells were nucleofected using Lonza P3 solution and program CA-137 with RNP complexes of 61 pmoles each of Alt-R^™^ S.p. HiFi and dCas9 Nucleases from IDT and 175 pmoles Synthego sgRNA targeting GACGCAAGTACCTTAGAATT plus 120 pmoles of IDT Ultramer TARDBP K181E editing oligo, 5’-AGCGACATATGATAGATGGACGATGGTGTGACTGCAAACTTCCTAATTCTGAGGTACTTGCGTCTGTGCTTTGGGAATTTTTGCCAACAAACTTCCTTAG-3’. Cells were plated on hrLaminin-521 coated plates in StemFlex medium supplemented with 1x RevitaCell and 1 μM IDT HDR Enhancer V2 and incubated at 32 °C. Medium was changed to StemFlex supplemented with 0.5X RevitaCell after 24 hours and to StemFlex after 48 hours. On the third day, single cells were sorted with a BD FACSMelody cell sorter at 1 cell per well to a Corning^®^ Matrigel^®^ Matrix coated 96-well plate with StemFlex medium supplemented with STEMCELL Technologies CloneR^™^2. Incubation temperature was shifted back to 37 °C and medium shifted to StemFlex by replacing 1/2 volume every other day. gDNA was isolated from expanded clones using DNAdvance on a Biomek4000. The target region was PCR amplified with 5’-TGGGAATGGAGTGTGTGAGT-3’ and 5’-TTCTCTTTTCCATCCATAGCGA-3’ primers using Phusion^®^ Hot Start Flex DNA Polymerase. Product was purified with 0.8X AMPure XP beads and sequenced to identify both homozygous and heterozygous clones.

### Differentiation of iPSCs into forebrain neurons

The procedure was reported previously (24). Briefly, the procedure for inducing neuronal differentiation of iPSCs using an induction medium (IM-N2) and a neuronal culture medium (CM). IM-N2 is prepared with Knockout DMEM/F12, N2 supplement, NEAA, Gluta-MAX, Chroman I, and doxycycline. On Day 0, iPSCs were observed for confluency, dissociated with Accutase, centrifuged, and resuspended in IM-N2 with Chroman I, and then seeded into Matrigel-coated plates. Over the next four days, cells were monitored microscopically for neurite extensions while media containing doxycycline is refreshed daily. By Day 4, cells exhibit neurite growth and are ready for replating onto poly-L-ornithine (PLO)-coated dishes, prepared in advance by coating with PLO solution, incubating, washing, and drying. Replated cells were cultured in CM comprising BrainPhys medium, B27+ supplement, neurotrophic factors (GDNF, BDNF, NT-3), laminin, and doxycycline, to promote neuronal maturation.

### Differentiation of iPSCs into forebrain organoids

3D culture and organoid growth were performed as previously described (25). In brief, iPSCs grown on matrigel were dissociated into single cell suspension by Versene solution (ThermoFisher) and seeded into a 12-well Aggrewell plate (Stemcell Technologies) at 4,000 cells/well. Next day, spheroids were transferred to an ultralow attachment plate (Corning) containing phase I medium: DMEM/F12, 20% Gibco KnockOut Serum Replacement, 1X Glutamax, 1X MEM Nonessential Amino Acid, 55 μM β-Mercaptoethanol, 1X Pen/Strep, 2 μM Dorsomorphine, 2 μM A83–01. After 5 days, medium was switched to phase II medium: DMEM/F12, 1X N-2 Supplement, 1X Glutamax, 1X MEM Nonessential Amino Acid solution, 1X Pen/Strep, 4 ng/mL WNT3a, 1 μM CHIR-99021, 1 μM SB-431542. On day 7, spheroids were embedded into matrigel and allowed to continue growing for 7 more days. On day 14, individual spheroids were manually freed from the matrigel and transferred to a SpinOmega bioreactor spinning at 120 RMP with phase III media: DMEM/F12, 1X N-2 Supplement, 1X B-27 Supplement, 1X Glutamax, 1X MEM Nonessential Amino Acid, 55 μM 2-Mercaptoethanol, 1X Pen/Strep, 2.5 μg/mL insulin. 50 days later, the medium was switched to final differentiation medium: Neurobasal medium, 1X B-27 Supplement, 1X Glutamax, 55 μM 2-Mercaptoethanol, 1X Pen/Strep, 0.2 mM Ascorbic acid, 0.5 mM cAMP, 20 ng/mL brain-derived neurotrophic factor (BDNF), 20 ng/mL glial-derived neurotrophic factor (GDNF). 107-day-old organoids were dissociated into single cells using a 50:50 mixed of Accutase and 0.25% Trypsin with DNase I (1 mg/mL) and plated onto chambered glass slides pretreated with 1% Matrigel (Corning, Inc). Alternatively, organoids were fixed and sectioned for immunostaining (see below). For drug treatment, forebrain organoids (87 Day) were transferred to a 12-well plate on an orbital shaker (120 RPM) and treated with 20 nM KPT276 in final differentiation medium. Medium was changed every other day (Dexoregen, Inc). The organoids were harvested and frozen after 35 days of treatment for sectioning or RNAseq analysis.

### Tissue preparation, immunoblotting, and immunostaining

Organoids were fixed in 4% paraformaldehyde for 1 hour at room temperature, washed with PBS, and immersed in 15% sucrose/PBS solution overnight. Subsequently, organoids were embedded in O.C.T. compound (Sakura) in a plastic mold and frozen down in an ultralow freezer. Embedded organoids were sliced onto glass slides using a Cryostat (Leica). Slides were rinsed with PBS, permeabilized with 0.5% Triton-X/PBS solution for 1 hour at room temperature and blocked using 1% donkey serum in 0.1% Tween-20/PBS solution for 1 hour. Primary antibodies, chicken anti-TUJ1 (Abcam, ab41489,1/1000 dilution), rabbit anti-TDP-43 (10782-2-AP, 1/1000 dilution), mouse anti-phospho-TDP-43 (Cosmobio, CAC-TIP-PTD-M01A, 1/500 dilution), rabbit anti-cleaved caspase 3 (Cell signaling, 9664, 1/1000 dilution), were added to the slides and the slides were incubated in a humidified chamber at 4 °C overnight. After several washes in 0.1% Tween-20/PBS solution, DAPI (Sigma) or secondary antibodies, donkey anti-rabbit, anti-mouse, and anti-chicken (Jackson ImmunoResearch), diluted 1:1000 in blocking solution, were added to the slides. After 1 hour of incubation at room temperature, the slides were washed and mounted in antifade mounting solution (Fisher scientific).

For immunoblotting analyses, cells or organoids were first solubilized in NP40 lysis buffer containing 25 mM of Tris-HCl, pH 7.4, 150 mM of NaCl, 2 mM of MgCl_2_, 2 mM of KCl, and 0.5% of NP40, 1mM DTT and a protease inhibitor cocktail. After centrifugation at 20,000 g for 5 min, cleared supernatants were analyzed by immunoblotting with TDP-43 antibody (Proteintech, 10782–2-AP) and phospo-TDP-43 antibody (Cosmobio, TIP-PTG-M01A) at 1:500 dilution.

### Image acquisition and processing

Fluorescence confocal images were acquired using a Nikon CSU-W1 SoRa microscope equipped with temperature and CO_2_ control enclosures. Image reconstructions and analyses, including 3D and time lapse visualizations, were performed using Imaris software (licensed to NIH) or Image J. Fluorescence intensity was quantified using the open-source Fiji/Image J software. Images of randomly selected fields were split into individual channels. A consistent threshold method was applied to individual channel. The particle analysis function was used to automatically identify all other fluorescence structures for size and intensity measurement. Statistical analyses were conducted using Excel (for Student’s t-test) or GraphPad Prism versions 8.0 and 9.0. P-values were calculated using Studenťs t-test in Excel or one-way ANOVA in GraphPad Prism. Curve fitting, including linear and nonlinear models, as well as IC_50_ calculations, were also performed using GraphPad Prism versions 8.0 and 9.0.

### Single-cell RNAseq cDNA library preparation

We mixed 30 organoids from multiple clones, two independent batches (15/batch) for scRNAseq. Single cells were captured and barcoded using the 10X Chromium platform (10X Genomics), and scRNA-seq libraries were prepared according to the Chromium Single Cell 3 Reagent Kits User Guide (v3). Gel Bead-In Emulsions (GEMs) were generated using single-cell preparations and processed with a 10X Chromium Controller. Following GEM reverse transcription and cleanup, mRNA from uniquely barcoded cells was converted into complementary DNA (cDNA), which was amplified to generate sufficient mass for library construction. The amplified cDNA underwent enzymatic fragmentation, size selection (targeting an insertion size of approximately 350 bp), end-repair, A-tailing, adaptor ligation, and dual-index PCR to construct the single-cell 3 gene expression libraries. Quality control was performed using Agilent Bioanalyzer High Sensitivity DNA chips, and libraries were pooled to ensure a similar number of reads from each single cell before sequencing on the NovaSeq S4 (Novogen Inc).

### Single-cell RNA-seq data processing

Reads from scRNA-seq were mapped to human transcriptome (GRCh38) using Cell Ranger software (10x Genomics) with the default parameters for each sample separately. Genes were retained with expressing in more than 5 cells. Cells with less than 200 detected genes were excluded. We also performed additional quality control steps including removing doublets (according to the multiplet rate provided by 10x Genomics based on the number of cells loaded and recovered) and filtering cells with the percentage of mitochondrial genes (≥ 10%). R package DoubletFinder (v2.0.4) was used to detect doublets with default parameters. A total of 16,549 cells passed the quality control in WT and K181E/K181E organoids. ‘SCTransform’ method from R package Seurat (v 5.1.0) was used to perform data normalization and scaling based on the top 2000 highly variable genes. The first 15 PCs were selected for tSNE and clustering analysis with default parameters after data reduction with PCA. Different subclusters of single cells were revealed from the tSNE plot.

### Cell type annotation

Differentially expressed genes (DEGs) for each cluster were first identified by ‘FindAllMarkers’ from Seurat (v 5.1.0) using the following criteria: adjusted p-value < 0.05, ≥10% of cells expressing the gene, and log_2_ fold change ≥ 1 (Supplementary Table S1). These marker genes were analyzed using the ACT web server (http://biocc.hrbmu.edu.cn/ACT/), which employs a weighted and integrated gene set enrichment (WISE) method and knowledge-based resources for initial cell type characterization. The results were then manually refined based on the classical markers of different cell type, and the finalized cluster annotations with detailed gene marker expression are included in Supplementary Table S1.

### Construction of single cell trajectory

R package Monocle3 (v1.3.7) was used to learn the trajectory graph based on scRNA-seq data. Top 2000 most variable genes from ‘FindVariableFeatures’ and top 15 PCs were used to perform Uniform Manifold Approximation and Projection (UMAP) and Leiden clustering. The trajectory graph for single cells was learnt using R function (learn_graph) with default parameters. We utilized R package CytoTRACE2 (v1.0.0) to calculate the developmental potential score for single cell. The count matrix was set as input with default parameters.

### REIN score calculation

We used the expression of REIN signature genes to examine the enrichment differences of REIN phenotype in ALS/FTD patients (26). REIN related genes were defined using R function “FindVariableFeatures” from Seurat (v5.1.0). Genes expressed in ≥ 20% of REIN with adjusted P-values < 0.05 and foldchange ≥ 4 were considered as REIN related genes (Supplementary Table S2). REIN score was calculated as the average expression of the identified REIN related genes.

### Pathway enrichment analysis

Gene enriched biological pathways (Gene Ontology) were detected using R package ClusterProfiler (v4.14.0) (27) with cutoff of P-value < 0.05. R package ggplot2 (v3.5.1) was used to plot the GO figures. The enriched KEGG pathways were obtained using Enrichr (https://maayanlab.cloud/Enrichr/).

### Cell-cell communication analysis

R package CellChat (v2.1.2) (28) was used to detect significant cell-cell communication signaling among different cell types. Secreted signaling in CellChat database was selected for downstream analysis. ‘trimean’ method was used to calculate communication probability of ligand-receptor signals. Ligand-receptor signals detected in at least ten cells were retained for downstream analysis.

### Gene Ontology (GO) analysis

Gene enriched biological pathways were detected using R package ClusterProfiler (v4.14.0) (27) with cutoff of P-value < 0.05. R package ggplot2 (v3.5.1) was used to plot the GO figures.

### eCLIPseq library preparation and data analysis

eCLIP was performed by Eclipsebio according to the published single-end seCLIP protocol with the following modifications (29). Approximately 40 mg of sample from each condition was cryogrinded and UV crosslinked at 400 mJoules/cm^2^ twice with 254 nm radiation. Cryoground tissue was then stored until use at −80 °C. Tissue aliquots were lysed using 750 μL of eCLIP lysis mix with a modified composition of 6 μL of a proteinase Inhibitor Cocktail and 20 μL of murine RNase inhibitor. Samples were then subjected to two rounds of sonication for 4 minutes with 30 second ON/OFF at 75% amplitude. A pre-validated anti-TDP-43 antibody (Eclipsebio) was then coupled to IgG Dynabeads (ThermoFisher), added to lysate, and incubated overnight at 4°C. Prior to immunoprecipitation, 2% of the sample was taken as the paired input sample, with the remainder magnetically separated and washed with eCLIP high stringency wash buffers. IP and input samples were cut from the membrane at the relative band size to 75kDa above. RNA adapter ligation, IP-western, reverse transcription, DNA adapter ligation, and PCR amplification were performed as previously described (29).

The eCLIP cDNA adapter contains a sequence of 10 random nucleotides at the 5’ end. This random sequence serves as a unique molecular identifier (UMI) after sequencing primers are ligated to the 3’ end of cDNA molecules. Therefore, eCLIP reads begin with the UMI and, in the first step of analysis, UMIs were pruned from read sequences using umi_tools (v0.5.1) (30). UMI sequences were saved by incorporating them into the read names in the FASTQ files to be utilized in subsequent analysis steps. Next, 3’-adapters were trimmed from reads using cutadapt (v2.7), and reads shorter than 18 bp in length were removed. Reads were then mapped to a database of human repetitive elements and rRNA sequences compiled from Dfam (31) and Genbank. All non-repeat mapped reads were mapped to the human genome (hg38) using STAR (v2.6.0c) (32). PCR duplicates were removed using umi_tools (v0.5.1) by utilizing UMI sequences from the read names and mapping positions. Peaks were identified within eCLIP samples using the peak caller CLIPper (33).

For each peak, IP versus input fold enrichment values were calculated as a ratio of counts of reads overlapping the peak region in the IP and the input samples (read counts in each sample were normalized against the total number of reads in the sample after PCR duplicate removal). A P-value was calculated for each peak by the Yates’ Chi-Square test, or Fisher Exact Test if the observed or expected read number was below 5. Comparison of different sample conditions was evaluated in the same manner as IP versus input enrichment; for each peak called in IP libraries of one sample type we calculated enrichment and P-values relative to normalized counts of reads overlapping these peaks in another sample type. Peaks were annotated using transcript information from GENCODE (34) with the following priority hierarchy to define the final annotation of overlapping features: protein coding transcript (CDS, UTRs, intron), followed by non-coding transcripts (exon, intron). HOMER de novo motif analysis pipeline was used to identify significantly enriched binding motifs of TDP-43 in each sample condition (eCLIPSEBIO) (35).

### Bulk RNA-seq library preparation and data analysis

800 ng of total RNA samples to generate the library with the NEBNext rRNA Depletion Kit v2 (Human/mouse/rat) (NEB #E7405), NEBNext Ultra II Directional RNA Library Prep Kit for Illumina (NEB #E7760) and NEBNext^®^ Multiplex Oligos for Illumina^®^ (96 Unique Dual Index Primer Pairs, NEB, E6440) as described in the manufacturer’s manual. Sequencing carried out in the Illumina NextSeq2000 instrument with 101×101 pair end configuration by NCI genomic core.

Adapters were trimmed using Cutadapt (v4.7), and reads were aligned to the GRCh38 genome build with STAR (v2.7.11b) using gene annotations from GENCODE v42. Gene expression was quantified with HTSeq (v2.0.7) (36), utilizing the same gene annotations. For differential gene expression analysis, all samples were processed uniformly following the standard DESeq2 (37) workflow. Differential expression was defined based on an adjusted P-value threshold of <0.05 and |log2 fold change (log_2_FC) > 1. To identify gene expression rescued by exportin-1 inhibitor treatment, the fold change of expression level should be less than without inhibitor treatment, with P-value < 0.05.

### RNA Splicing Analysis

For differential splicing analysis, STAR-aligned BAM files from RNA-seq dataset were used as input for rMATS (v4.1.2) (38) with the GRCh38 reference genome. A ΔΨ (percent spliced in) threshold of 10% was applied to identify significant changes between groups. Junction files from rMATS were further analyzed using custom Python scripts to compute Ψ values for each splicing junction. ΔΨ values were calculated as the mean Ψ of three biological replicates. Cryptic splicing events were identified as splicing junctions that met the following criteria: Ψ < 5% in control samples, ΔΨ > 10%, and the junction was not annotated in GENCODE v42.

### Whole exome sequencing and off-target analysis

10 ug of genomic DNA was extracted from WT stem cell and K181E/K181E stem cell, then sent out for whole exosome sequencing provided by Azenta Life Sciences. Briefly, Sequencing adapters and low-quality bases in raw reads were trimmed using Trimmomatic 0.39. Cleaned reads were then aligned to the Homo sapiens GRCh38 reference genome using Sentieon 202308. After sorting the alignments, PCR and optical duplicates were flagged and removed. Single-nucleotide variants (SNVs) and small insertions and deletions (INDELs) were identified using Sentieon 202308 with the DNAscope algorithm. Variant call format (VCF) files generated by the pipeline were subsequently normalized with bcftools 1.13 by left-aligning INDELs and decomposing multiallelic sites into multiple records. Top 20 predicted off target sequences were obtained from CRISPOR and further cross-checked in the processed whole exosome sequencing files (Table S6).

### Protein structural simulation

WT PRDM2 and PRDM2+CE protein structures were simulated by AlphaFold Server(https://alphafoldserver.com/) and aligned by PyMOL.

### Statistic and reproducibility

All statistical analyses were conducted with GraphPad Prism v10 or Excel. Statistical methods and the number of cells or brain organoid samples (N) are indicated in figure legends or shown in figures as individual data point. Biological repeats (n) are specified in figure legends. We did not predetermine sample size. The sample sizes are consistent with similar studies reported in the literature. No biological repeat was excluded from the analyses. For individual cell analyses, a few data points (less than 2%) outside of 1.5 times the interquartile range were considered as outliers and were excluded. All experiments were repeated at least twice with individual data point labeled in figures unless specified. The immunoblotting is representative of similar results from at least two independent biological replicates unless specified in figure legends. For imaging analyses, cells in randomly selected fields were analyzed. The researchers were not blinded. All iPSC cell lines were derived from a ADRDs genetic risk-free clone, which was initially obtained from a male. Figures were prepared using ImageJ 1.54f, Imaris 9.9.0, Adobe Photoshop v25.12.1, and Adobe Illustrator 28.7.4.

## Results

### A forebrain brain organoid model of TDP-43 proteinopathies

To model TDP-43-associated proteinopathies, we used CRISPR-mediated gene editing to introduce the ALS/FTD-associated K181E mutation at the endogenous *TARDBP* locus in a well-characterized dementia genetic risk-free iPSC line followed by extensive whole exome sequencing to rule out CRISPR mediated off target effect (see method and table S6). Genomic sequencing identified several K181E heterozygous and homozygous (HM) knock-in (KI) clones (Supplementary Fig. S1a). We selected clones with normal karyotypes and confirmed pluripotency (Supplementary Fig. S1b) and transduced them with a lentiviral vector that enabled their differentiation into excitatory neurons (Exci-neurons) via tetracycline-induced expression of neurogenin-2 (NGN2) (39). We then used immunoblotting to examine hyperphosphorylated TDP-43 (p-TDP-43) at Ser409/410, a hallmark of TDP-43 proteinopathies by a widely used antibody (40–43), but failed to detect it in differentiated neurons under normal growth conditions regardless of the genotype (Supplementary Fig. S1c, d); Only after treatment with a proteasome inhibitor (MG132), did we observe a small amount of p-TDP-43 in both wild-type (WT) and K181E neurons, suggesting that p-TDP-43 generated from WT and K181E TDP-43 is rapidly degraded by the proteasome and that this cell model does not phenocopy TDP-43 proteinopathies.

**Figure 1:**
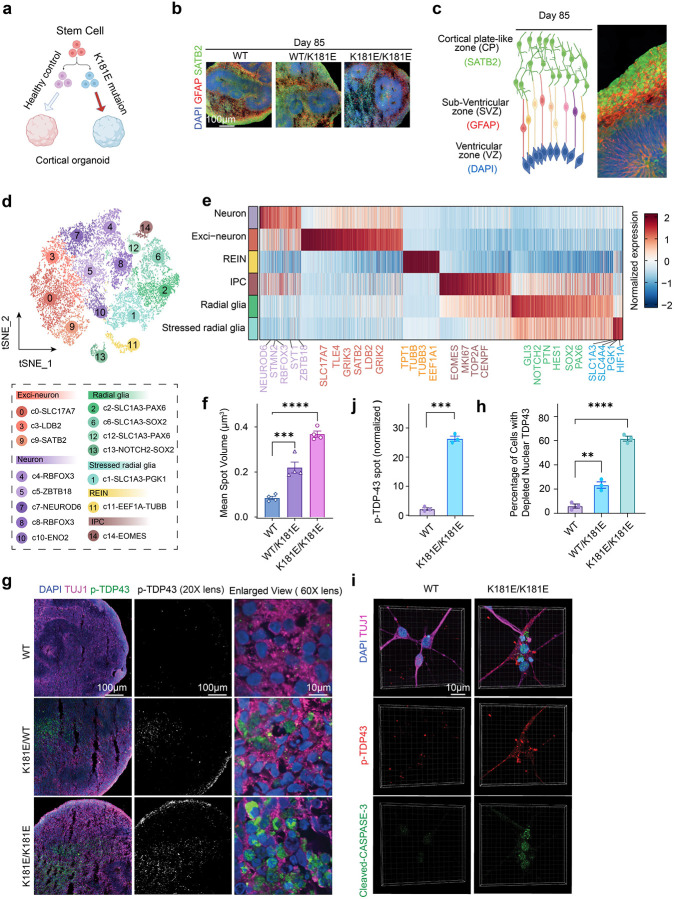
A forebrain brain organoid model of TDP-43 proteinopathies. **(a)** Schematic representation of the development of forebrain brain organoids. **(b)** Panels show immunostaining of organoid sections with antibodies against the indicated neuronal markers at day 85 (n= 3–5 organoids, 2–3 iPSC clones, two individual batches). **(c)** A representative immunofluorescence image (right) demonstrats the formation of a forebrain-like structure in organoids at day 85, as illustrated in the schematic (left). **(d)** T-distributed Stochastic Neighbor Embedding (**t-SNE**) of 16,549 single cells in WT and K181E/K181E organoids, colored by cell clusters. Color code and cluster ID are shown (below). **(e)** Heatmap showing the expression of cell type-specific genes (log2 FC≥0.5 & adjusted p-value < 0.05 & percentage of expressed cells ≥ 10%). Representative gene markers are grouped and colored by corresponding cell types. **(f, g, h)** Phosphorylated TDP-43 accumulates in K181E mutant organoids. **(f)** Quantification of phosphorylated TDP-43 (p-TDP43) mean spot volume in 3D fluorescence images of immuno-stained organoids with the indicated genotypes. Error bars indicate mean ± s.e.m., **** p < 0.0001, *** p < 0.001 by one-way ANOVA, n=4 organoids. Representative confocal images are shown in **(g)**. Scale bar, 10 μm, n= 3–5 organoids, 2–3 iPSC clones, two individual batches. (**h**) Quantification of cells with depleted nuclear TDP43 with the indicated genotypes. Error bars indicate mean ± s.e.m., **** p < 0.0001, ** p < 0.01 by one-way ANOVA, n=3 organoids. **(i)** Representative fluorescence images of neurons dissociated from mature organoids at Day 107 and cultured as isolated neuronal cells before fixing and immunostaining at Day 127. Neuronal marker TUJ1 (magenta), p-TDP43 (red), apoptosis marker cleaved Caspase-3 (green), and DAPI (blue) are shown. n= 3–5 organoids, 2–3 iPSC clones, two individual batches. (**j**) Quantification of normalized p-TDP43 spot number per cell in 2D fluorescence images of disassociated cells with the indicated genotypes. Error bars indicate mean ± s.e.m., *** p < 0.001 by unpaired Student’s t-test, n=3 organoids.

To generate a better TDP-43 proteinopathy model, we used a growth factor-based system to differentiate and grew these iPSC cells from multiple clones into 3D organoids (Fig. 1a) because organoids can reconstruct cell diversity and cell-cell communications essential for pathogenesis. To this end, we first screened and identified a ROCK2 inhibitor as a critical element that supported forebrain-like growth during the early phase of differentiation, particularly for cells bearing the disease mutation. We then maintained the cultures for 85 days, at which point, we used immunostaining to confirm the robust expression of key markers including SATB2 (Exci-neuron) and GFAP (glial marker) (Fig. 1b). The staining also revealed the formation of distinct forebrain layers, mirroring the laminar organization of the brain (Fig. 1c).

Single-cell transcriptomic profiling of pooled organoids from two independent batches (Supplementary Fig. S1e) revealed various brain cell types including radial glia, stressed radial glia, intermediate progenitor cells (IPC), and Exci-neurons (Fig. 1d, e, Supplementary Fig. S2a–e, Supplementary Table S1), as reported previously (44), indicating the formation of complex cellular structures in these organoids. The presence of both neuronal and radial glia underscores the physiological relevance of our model because abnormal neuron-glia interactions are known to induce neurodegeneration (16).

We sectioned these organoids and stained them with p-TDP-43 antibodies. Noticeably, while minimal p-TDP-43 signal was detected in WT organoids, we saw some p-TDP-43 signal in heterozygous organoids and significantly more p-TDP-43 in K181E homozygous (HM) organoids suggesting a dose-dependent like effect (see discussion). K181E HM organoids had a significant percentage of cells depleted of nuclear TDP43 (Fig. 1f–h). Co-immunostaining using antibodies against the neuron specific protein TuJ1 showed substantial co-localization of p-TDP-43 with TuJ1, suggesting possible cytoplasmic accumulation in neurons in HM K181E organoids (Fig. 1g, right panels). Because HM K181E organoids had stronger TDP-43 proteinopathy phenotypes, we focused our study on HM K181E organoids.

p-TDP43 signal in brain organoids displays large patch-like puncta similar to that found in cortex tissue (45), obscuring its precise subcellular localization. To further confirm the cytosolic localization of p-TDP-43, we dissociated cells from the organoids and cultured them in monolayer. This allowed cell visualization with high resolution. As reported previously (12), most p-TDP-43 signals were detected in the cytoplasm in HM K181E cells (Fig. 1i, j). Moreover, staining with antibodies recognizing cleaved Caspase-3 detected Caspase-3 activation in some p-TDP-43 positive cells (Fig. 1i), consistent with the reported association of neurotoxicity with TDP-43 proteinopathy (46). Thus, the 3D TDP-43 K181E organoid model replicates key features of TDP-43 proteinopathy including endogenous TDP-43 cytoplasmic aggregation and associated neuronal cell death. Importantly, these phenotypes were developed without TDP-43 overexpression or exposing cells to an external stressor.

### Transcriptomic characterization of TDP-43 K181E organoids

**Figure 2:**
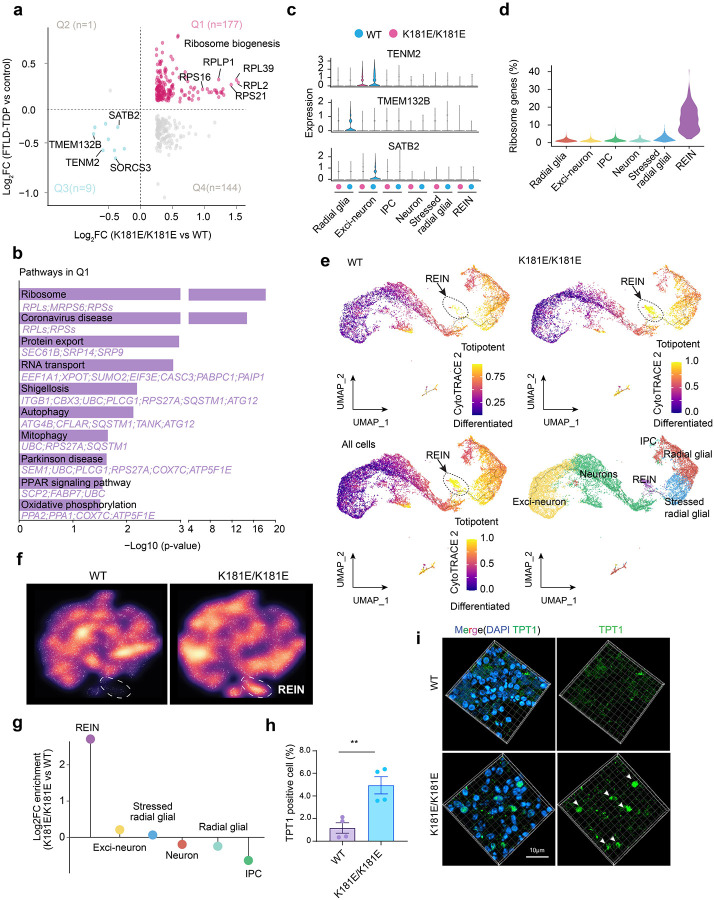
Single cell transcriptomic characterization of homozygous TDP-43 K181E organoids. **(a)** Comparative analysis of gene expression changes in K181E/K181E forebrain brain organoids (K181E/K181E) vs. wild-type (WT) forebrain brain organoids (x axis), and in ALS/FTD patient forebrain brains with confirmed TDP-43 aggregates (FTLD-TDP) vs. normal patient forebrain brains (control) (y axis). A total of 177 commonly upregulated genes (Q1, magenta) and 9 commonly downregulated genes (Q3, blue) were identified. **(b)** Top enriched KEGG pathways of the commonly upregulated genes in **(a)** (n=177) highlight the upregulation of protein biogenesis and quality control in mutant organoids. **(c)** Violin plots comparing the expression levels of TENM2, SATB2, and TMEM132B for the indicated cell types in WT and K181E/K181E forebrain brain organoids. **(d)** Violin plot showing the percentage of ribosome-related genes enriched in the indicated cell types. **(e)** Trajectory analysis of cell differentiation state in WT (top left), K181E/K181E (top right), WT and K181E/K181E (All cells, bottom left) organoids. Cells are colored by CytoTRACE scores. Higher score indicates more undifferentiated state. Ribo-enriched immature neurons (REIN) are highlighted by dashed lines. Bottom right: Uniform Manifold Approximation and Projection (UMAP) embeddings of single cells in WT and K181E/K181E organoids, colored by cell types. The black lines label the structure of the graph. **(f)** Cell density plots showing the accumulation of Ribo-enriched immature neurons (REIN) (indicated by dashed lines) in K181E/K181E forebrain brain organoids. **(g)** Log_2_ FC of cell type enrichment in K181E/K181E forebrain organoids compared to WT organoids. **(h)** Quantification of TPT1 expression determined by immunostaining in **(i)** in organoids of the indicated genotypes. Error bars indicate mean ± s.e.m., ** p < 0.01 unpaired Student’s t-test, n=4 organoids. **(i)** Reconstructed 3D confocal fluorescence images of immuno-stained organoid sections show increased expression of TPT1 (green) in K181E/K181E organoids. Organoids were co-stained with DAPI (blue) to label nuclei. Scale bar, 10 μm, n= 3–5 organoids, 2–3 iPSC clones, two individual batches.

To evaluate the clinical relevance of our model, we first compared the overall dysregulated gene expression in HM K181E organoids with genes dysregulated in postmortem brain samples of dementia patients with confirmed TDP-43 proteinopathy (26). Strikingly, among the 178 genes upregulated in patient samples (26), 177 (99.4%) were also upregulated in HM K181E organoids when compared to WT organoids. By contrast, among the 153 genes downregulated in patients, only 9 (5.9%) were similarly reduced in HM K181E organoids (Fig. 2a, Supplementary Table S2). Gene Ontology (GO) analysis of the 177 upregulated genes (Q1 in Fig. 2a) highlighted a significant enrichment in stress adaptation pathways such as protein translation, ribosomal biogenesis, and protein quality control (Fig. 2b). By contrast, genes down-regulated specifically in patients are mostly involved in synaptic connectivity regulations (e.g. glutamatergic synapse and gap junction) (Supplementary Fig. 3a). These functions could be influenced by aging, inter-tissue communications, and other factors missing in organoids. We therefore conclude that our 3D organoid model recapitulates key cellular responses to TDP-43 proteinopathy in patients.

Cell type specific gene expression analysis showed that the commonly downregulated genes such as SATB2 and TENM2 had the most significantly reduced expression in Exci-neurons, while TMEM132B was more dramatically downregulated in radial glia in HM K181E mutant organoids (Fig. 2c). These genes have key functions in forebrain neuron development and synaptic plasticity (47–50). Thus, their suppression may contribute to the abnormal brain functions in patients with TDP-43 proteinopathies.

Notably, scRNA-seq also revealed the upregulation of many ribosomal genes in a cluster of cells (~5%) in HM K181E organoids (Fig. 2d–g, Supplementary Table S1). The high expression of SYT1 gene in this group indicated their neuronal fate. CytoTRACE2-based differentiation potency analysis suggested that this cell type is least differentiated compared to others (Fig. 2e). We name these cells Ribo-Enriched Immature Neurons (REINs). Immunostaining of organoid sections using antibodies recognizing TPT1, a nuclear protein upregulated in REINs (Fig. 1e), confirmed the enrichment of this novel cell type in HM TDP-43 mutant organoids (Fig. 2h, i). Additional bioinformatic analyses of published patient RNAseq data revealed a similar enrichment of REIN signature genes in ALS and FTD patients (51), particularly in a population of Exci-neurons in FTD patients (Supplementary Fig. S3b). The appearance of the REIN population might be a facet of the stress adaptation response to TDP-43 proteinopathy. Together, these results corroborate recent findings that implicate abnormal protein translation in TDP-43 proteinopathies and related diseases (52).

### Single cell analysis reveals neuroinflammatory signatures in TDP-43 K181E organoids

**Figure 3:**
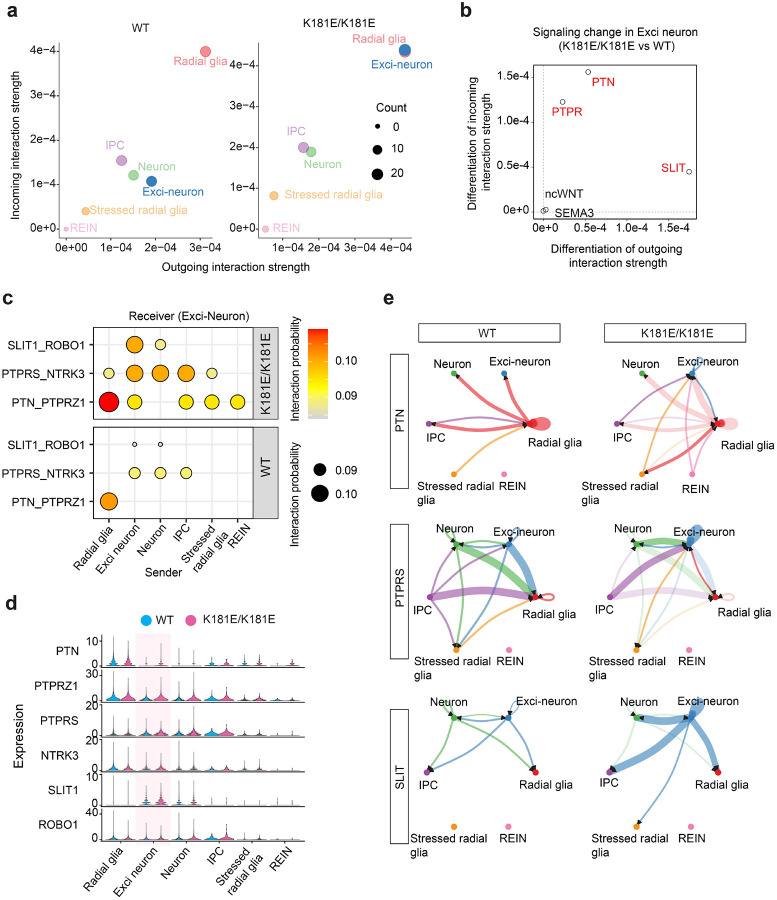
Single cell transcriptomic analysis reveals dysregulated cell-cell communications in TDP-43 K181E organoids **(a)** Scatter plots showing the relative sender and receiver strength in WT (left) and K181E/K181E (right) forebrain organoids. x-axis and y-axis indicate the total outgoing or incoming interaction strength associated with each cell type. Dot size indicates the number of inferred ligand-receptor pairs associated with each cell type. Different cell types are color-coded. **(b)** Altered signaling dynamics in Exci-neurons in K181E/K181E organoids compared to WT organoids. Pathways significantly increased in K181E/K181E organoids were colored in red. **(c)** The relative contribution of the ligand-receptor pairs to the increased signaling activity in K181E/K181E organoids as shown in **(b)** between the indicated cell types as sender and Exci-neuron as receiver. The relative Interaction strength/probability is indicated by both color and dot size. **(d)** Violin plots showing the relative expression of the indicated genes in different cell types in WT and K181E/K181E forebrain organoids. Note that the most significant changes were observed in Exci-neurons (shaded). **(e)** Circle plots showing interaction strength of PTN (top), PTPR (middle), and SLIT (bottom) between different cell types in WT (left) and K181E/K181E (right) organoids. Node size and edge size indicate the corresponding interaction strength. Arrows indicate the signaling direction.

To elucidate if TDP-43 K181E KI affects cell-cell communications in differentiated organoids, we conducted CellChat analysis (28) using our single-cell transcriptomic data. We first compared outgoing and incoming signals for different cell types between HM K181E mutant and WT organoids. Our analysis suggested that in WT organoids, while radical glia have high outgoing and incoming signals (Fig. 3a, left panel), Exci-neurons only display intermediate levels of outgoing and incoming signals. By contrast, in HM K181E mutant organoids, exci-neurons display a drastic increase in both outgoing and incoming signals (Fig. 3a, right panel). Pathway analyses identified several signaling processes, represented by the PTN-PTPRZ1, PTPR-NTRK3, and SLIT1-ROBO1 ligand-receptor pairs, as key contributors to this disruption in Exci-neurons (Fig. 3b, c). Notably, the upregulated incoming signaling in Exci-neurons are accompanied by elevated expression of both ligands and receptors (Fig. 3d), suggesting a self-stimulated autocrine regulation. Among the three ligand-receptor pairs, the PTPRS-NTRK3 signaling axis was previously implicated in synaptic development and neuronal stress responses (53). Its activation may represent a compensatory mechanism that counteracts synaptic dysfunction and neuronal stress in mutant cells. However, prolonged activation of this pathway seems to inadvertently exacerbate synaptic instability to contribute to TDP-43-associated neurodegeneration, as suggested previously (54).

Additionally, we observed altered paracrine signals in these three pathways in HM K181E mutant organoids including de novo interactions via the PTN-PTPRZ1 axis between REINs, Radial glia and Exci-neurons (Fig. 3e). Immunostaining using PTPRZ1 antibodies confirmed the upregulation of PTPRZ1 expression in the forebrain layer of HM K181E organoids where Exci-neurons are enriched (Supplementary Fig. S4a, b). Since PTN and PTPRZ1 were known to activate the release of cytokines such as TNFα, IL6 and IL10β (55), these new interactions may promote neuroinflammation. Indeed, immunostaining with TNFα antibodies detected an upregulation of TNFα expression in HM K181E organoids (Supplementary Fig. 4c, d). Collectively, our findings reveal abnormal signaling networks and intercellular communications in mutant organoids that alter synaptic regulation and elicit neuroinflammation.

### Altered RNA recognition and processing in TDP-43 K181E organoids

**Figure 4:**
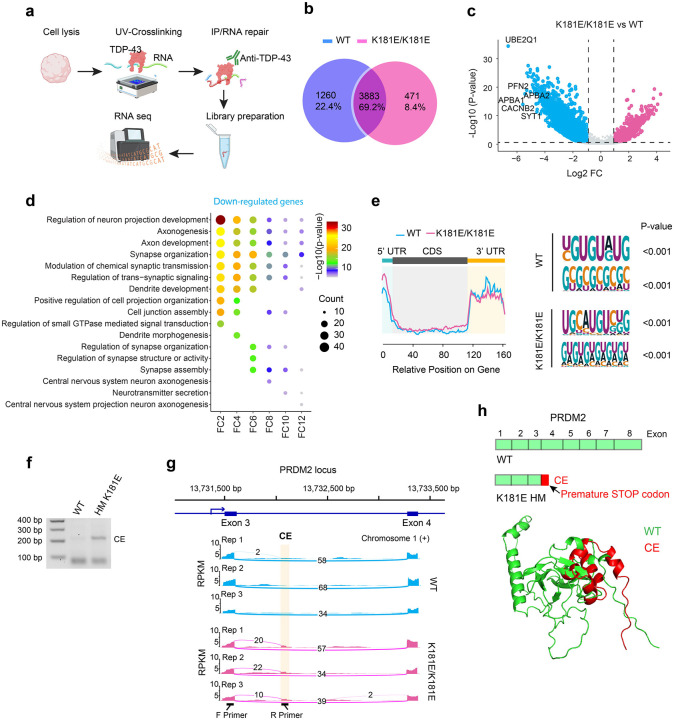
Altered RNA recognition and processing by the TDP-43 K181E mutant **(a)** Schematic of the eCLIP workflow. **(b)** A Venn diagram summarizes TDP-43-associated RNAs detected by eCLIP in WT and TDP-43 K181E/K181E organoids. **(c)** Volcano plot showing RNAs differentially bound by TDP-43 in K181E/K181E organoids compared to WT organoids. P-value < 0.05 and foldchange (FC) ≥2 was used to identify differentially upregulated (red) or downregulated (blue) binding sites in TDP-43 K181E/K181E organoids compared to WT organoids. **(d)** Pathway analysis of RNAs with reduced TDP-43 binding in TDP-43 K181E mutants compared to WT organoids. Genes were grouped by the indicated fold-change (FC) of reduced binding before pathway analysis. Colors represent the −log_10_ (P-value) of pathway enrichment. **(e)** Metagene analysis (left panel) reveals average number of peaks enriched in WT and the K181E/K181E mutant across different RNA regions (Y-axis) and peak’s relative position on gene (X-axis). Detected TDP43 RNA recognition motifs in WT and K181E/K181E mutant (right panel), p < 0.001 by Yates’s Chi-Square test. **(f)** RT-PCR ad gel electrophoresis confirms the presence of the predicted CE in the *PRDM2* transcript in K181E/K181E organoids. **(g)** Sashimi plot showing the inclusion of a cryptic exon (CE) in the *PRDM2* mRNA in K181E/K181E organoids in 3 replicates. **(h)** Schematic shows that the CE in *PRDM2* introduces an in-frame premature stop codon between exon 3 and exon 4 (top). The graph below shows an alpha-fold modeled structure of the translational product PRDM2* that contains the cryptic peptide (red) and its alignment with the structure of the corresponding WT PRDM2 N-terminal region.

Since TDP-43 is an RNA binding protein, we analyzed RNA binding and processing by TDP-43 in WT and HM TDP-43 K181E organoids using eCLIP, a crosslinking and immunoprecipitation-based RNAseq assay for capturing global RNA-protein interactions (Fig. 4a, Supplementary Fig. S5a) (29). The analysis identified 33,069 and 25,675 binding events for WT and K181E mutant, corresponding to 5,143 and 4,354 genes, respectively (Fig. 4b). Among these genes, 1,260 (22.4%) bound specifically to WT TDP-43, and 471 (~8%) bound to TDP-43 K181E only. While the majority (69.2%) of the identified RNAs bound to both WT and mutant TDP-43, their affinity to these proteins was different, with 3016 RNAs showing reduced and 893 RNAs showing increased binding to the K181E mutant (Fig. 4c). GO pathway analysis on RNAs with reduced affinity to TDP-43 K181E revealed that TDP-43 binding deficiency predominantly occurred in genes involved in synapse assembly and trans-synaptic signaling (Fig. 4d) and mRNA metabolism (Supplementary Fig. S5b, Supplementary Table S3). Thus, the disease mutation alters TDP-43’s RNA-binding landscape, affecting primarily RNA metabolism and synaptic communications.

Despite the differential RNA binding activity, sequence analysis showed that both WT and the K181E TDP-43 mutant bind predominantly to non-coding regions including introns and 5’- and 3’-UTRs, albeit with distinct sequence preference (Fig. 4e, Supplementary Table S4). This result suggests that the disease mutation disrupts TDP-43's canonical RNA-binding specificity, causing a global change in RNA binding.

Since TDP-43 is known to regulate RNA splicing, particularly in suppressing cryptic exon (CE) inclusion (9, 56–58), we investigated RNA splicing defects in HM TDP-43 K181E mutant organoids. To this end, we performed high reads RNAseq using bulk RNAs isolated from WT and K181E HM organoids and applied a well-established splicing analysis method named rMATS (38) to 500 million reads. This analysis identified 48 candidate RNA splicing events that might be altered in mutant organoids (Supplementary Table S5). Among them, 8 genes appeared to contain a CE in mutant organoids. As expected, we detected a cryptic splicing site in STMN2 (Supplementary Table S5), a well-established neuronal gene whose mis-splicing occurs in TDP-43-associated proteinopathies (59, 60).

We next investigated how RNA mis-splicing affect gene transcription products in mutant organoids. To this end, we conducted RT-PCR using a primer aligned to the predicted CE sequence and a primer in the preceding exon for each of the 8 candidates. The amplified DNA fragments were analyzed by electrophoresis and Sanger’s sequencing. Among the genes analyzed, the most significant change occurred in PRDM2 (Fig. 4f, Supplementary Fig. S5c), which encodes an epigenetic factor regulating cellular senescence (61). In HM K181E mutant organoids, ~14% PRDM2 mRNAs has a CE of 490 bp long, derived from an intron sequence between exon 3 and 4 (Fig. 4g). The mis-spliced *PRDM2* RNA bears an in-frame premature stop codon (Fig. 4h). Upon translation, it would generate a truncated PRDM2 mutant (PRDM2*) of 56 amino acids long, containing a 14 amino acid C-terminal cryptic peptide (NH_2_-VPDTLWTLSRYLNE-COOH) translated from the CE (Supplementary Fig. S5d, Fig. 4h). Although mass spectrometry did not detect this cryptic peptide in mutant organoids, we observed ~15% reduction in total PRDM2 protein level in mutant organoids after 135 days in culture (Supplementary Fig. 5e). The correlation between reduced protein level and amount of CE-bearing PRDM2 mRNA suggests that PRDM2* bearing the cryptic peptide may be unstable. Collectively, these results demonstrate that the K181E mutation alters the TDP-43 RNA-binding specificity, causing a major defect in RNA binding and splicing.

## Discussion

Our study establishes an iPSC-based forebrain brain organoid model carrying the ALS- and FTD-associated TDP-43 K181E mutation, which recapitulates key phenotypes of TDP-43 proteinopathies including endogenous TDP-43 cytoplasmic hyperphosphorylation, RNA dysregulation, disturbed protein homeostasis, neuroinflammation, and cell death. These phenotypes were not seen when the same iPSCs were differentiated into neurons in 2D culture. The fact that most genes upregulated in HM TDP-43 K181E organoids were similarly dysregulated in FTD patients underscores the physiological relevance of the model. The new model has several advantages that allow better phenocopying TDP-43 proteinopathies. In addition to a 3D laminar architecture resembling human forebrain brain structure, the organoids contain most brain cell types except for microglia, allowing complex intercellular communications essential for disease development. Consistent with this notion, CellChat analyses suggest extensive cell-cell communications within the neuronal populations and between distinct cell types in organoids. Notably, many interactions between Exci-neurons and other cell types are altered in mutant organoids due to altered expression of signaling molecules such as the SLIT1-ROBO1, PTN-PTPRZ1, and PTPR-NTRK3 ligand-receptor pairs. These abnormal cell-cell interactions may contribute to disrupted synaptic connectivity during disease progression (53, 55, 62).

The reconstitution of aberrant intercellular signaling in 3D organoid models underscores the complex interplays between distinct cell populations in disease progression. In this regard, restoring neuronal connectivity and reducing neuroinflammatory responses through targeted interventions may offer new therapeutic avenues. For instance, targeting the PTN-PTPRZ1 signaling axis, known to elicit pro-inflammatory responses, may provide additional benefit by attenuating neuroinflammation and preserving neuronal function.

Among the cell types found in the organoids, we noticed a previously uncharacterized immature neuronal population (REIN) with elevated expression of translation-related genes, which is only enriched in HM TDP-43 K181E organoids. scRNA-seq data from ALS and FTD patients supports the existence of an Exci-neuronal population in patients with a similar dysregulated REIN gene signature. In HM TDP-43 K181E organoids, these cells also exhibit aberrant interactions with Exci-neurons via the PTN-PTPRZ1 signaling axis, and therefore, may amplify TDP-43 proteinopathy defect via neuroinflammation induction. It is noteworthy that translation dysregulation has long been linked to ALS and FTD (52). Our findings suggest that translation dysregulation in a subpopulation of Exci-neurons may be a driving factor in ALS/FTD.

Another key finding is the identification of widespread RNA dysregulation driven by the TDP-43 K181E mutation. A previous study reported that this TDP-43 mutant fails to bind a canonical TDP-43-binding motif *in vitro* (19). Our unbiased eCLIP analysis reveals that the K181E mutation alters the TDP-43’s RNA-binding specificity, particularly in introns and 5’- and 3′-UTRs, leading to aberrant RNA processing in mutant organoids. Accordingly, we discovered many mis-splicing events in HM TDP-43 K181E organoids including CE inclusion in *PRDM2*. CE inclusion in *PRDM2* introduces a premature stop codon known to trigger non-sense-mediated RNA decay (NMD). Indeed, the expression of *PRDM2* is downregulated in FTD patients. Our finding that CE inclusion is a major splicing defect in TDP-43-associated proteinopathies corroborate recent reports (57, 58), underscoring the clinical relevance of our model. The change in TDP-43 binding to 5’- and 3’-UTRs may also affect protein translation given the established role of TDP-43 in translational regulation (63).

CE inclusion usually results in altered translational products with reduced function or a gain-of-toxic function (57). For *PRDM2*, the CE-containing mRNA encodes a truncated protein with a 14 amino acid carboxyl cryptic peptide derived from the CE. This and other abnormal translation products may have dominant gain-of-function activity that explains the dose effect of the K181E allele on TDP-43 proteinopathies. Future work is needed to determine the abundance of cryptic peptide-containing proteins in FTD and ALS patients, which may serve as biomarkers for disease diagnosis and therapeutic evaluation.

In summary, our study highlights the utility of human forebrain brain organoids as a platform for investigating the molecular linchpin of neurodegeneration. By capturing key aspects of patient-specific pathology including endogenous TDP-43 hyperphosphorylation, RNA deregulation, altered protein homeostasis, and aberrant intercellular signaling, our model bridges the gap between the current in vitro systems and in vivo disease mechanisms. Furthermore, the ability to dissect disease-relevant pathways at single-cell resolution unravels critical disease relevant cell-cell interactions and provides new opportunities for therapeutic development.

## Conclusions

A new human iPSC-derived forebrain organoid model carrying the TDP-43 K181E mutation displays important pathological features of TDP-43 proteinopathies including cytoplasmic TDP-43 aggregation, RNA dysregulation, neuronal loss, and activation of neuroinflammation. These phenotypes were not seen when the same cells were differentiated in 2-D cultures, demonstrating the advantage of 3-D organoid architecture in recapitulating the molecular and cellular complexity of TDP-43-associated neurodegeneration. Importantly, our multi-omics-based study has identified new pathological characteristics including an immature neuronal population with disrupted translation and aberrant signaling network, and altered splicing patterns in several target genes such as PRDM2. These findings link mutant TDP-43 activity directly to translational imbalance and impaired RNA metabolism in neurons. Together, these results provide mechanistic insight into how a single TDP-43 mutation drives neurodegeneration through coordinated disruption of RNA regulation, proteostasis, and cell-cell communication.

## Supplementary Material

Supplement 1

Supplement 2

Supplement 3

Supplement 4

Supplement 5

Supplement 6

Supplement 7

## Data Availability

Raw and processed data files of scRNA-seq, bulk RNA-seq and eCLIP-seq can be accessed from Sequence Read Archive (SRA), NCBI with Bioproject ID: PRJNA1195353, http://www.ncbi.nlm.nih.gov/bioproject/1195353. Code used in this project is available upon request. Whole exosome sequencing files and processed sequencing data files can be found at: https://zenodo.org/records/14291940?preview=1&token=eyJhbGciOiJIUzUxMiJ9.eyJpZCI6IjFlOWM5MzU3LTQyMzUtNGQzYy05MTY2LTlhNDk4NzI5NWU5OSIsImRhdGEiOnt9LCJyYW5kb20iOiIwNDYzYWY0NjJhODExZWQ5YzkzNzI4MzlhYmZmNjc4ZiJ9.crjqSUlB8r6LPGyMV8YaB9zSLzSdO1zE33tjjF1UVXaQqnWpy8ZOW9FU6VPrbNN5zyf2JXJOpfDteT_RaaZ8Zg

## Abbreviations

ALS: Amyotrophic lateral sclerosis
ANOVA: Analysis of variance
BDNF: Brain-derived neurotrophic factor
CE: Cryptic exon
CM: Neuronal culture medium
CRISPR: Clustered regularly interspaced short palindromic repeats
cDNA: Complementary deoxyribonucleic acid
DAPI: 4’,6-diamidino-2-phenylindole
DEG: Differentially expressed gene
DMEM: Dulbecco’s Modified Eagle Medium
eCLIP: Enhanced crosslinking and immunoprecipitation
Exci-neuron: Excitatory neuron
FBS: Fetal bovine serum
FTD: Frontotemporal dementia
FTLD-TDP: Frontotemporal lobar degeneration with TDP-43 pathology
GDNF: Glial-derived neurotrophic factor
GO: Gene Ontology
HM: Homozygous mutant
HR: Homology-directed repair
iPSC: Induced pluripotent stem cell
IM-N2: Induction medium containing N2 supplement
IPC: Intermediate progenitor cell
KI: Knock-in
LCD: Low-complexity domain
LLPS: Liquid–liquid phase separation
MG132: Carbobenzoxy-Leu-Leu-leucinal (proteasome inhibitor
NGN2: Neurogenin-2
NMD: Nonsense-mediated mRNA decay
PBS: Phosphate-buffered saline
PCA: Principal component analysis
PCR: Polymerase chain reaction
PLO: Poly-L-ornithine
PRDM2: PR domain zinc finger protein 2
PTN: Pleiotrophin
PTPRZ1: Receptor-type tyrosine-protein phosphatase zeta
qPCR: Quantitative polymerase chain reaction
RBP: RNA-binding protein
REIN: Ribo-enriched immature neuron
RIN: RNA integrity number
RNA-seq: RNA sequencing
RRM: RNA recognition motif
RT-PCR: Reverse transcription polymerase chain reaction
scRNA-seq: Single-cell RNA sequencing
TNFα: Tumor necrosis factor alpha
TDP-43: TAR DNA-binding protein 43
UMAP: Uniform manifold approximation and projection
UTR: Untranslated region
WT: Wild type
