# Supplementary material for: A human forebrain organoid model phenocopies dysregulated RNA and protein homeostasis in ALS/FTD-associated TDP-43 proteinopathies
Source: bioRxiv. 2025 Nov 10:2025.11.09.687455. Preprint. [Version 1] doi: 10.1101/2025.11.09.687455 (PMC12642313; doi:10.1101/2025.11.09.687455)
Supplement: Supplement 1 [file media-1.docx]

**
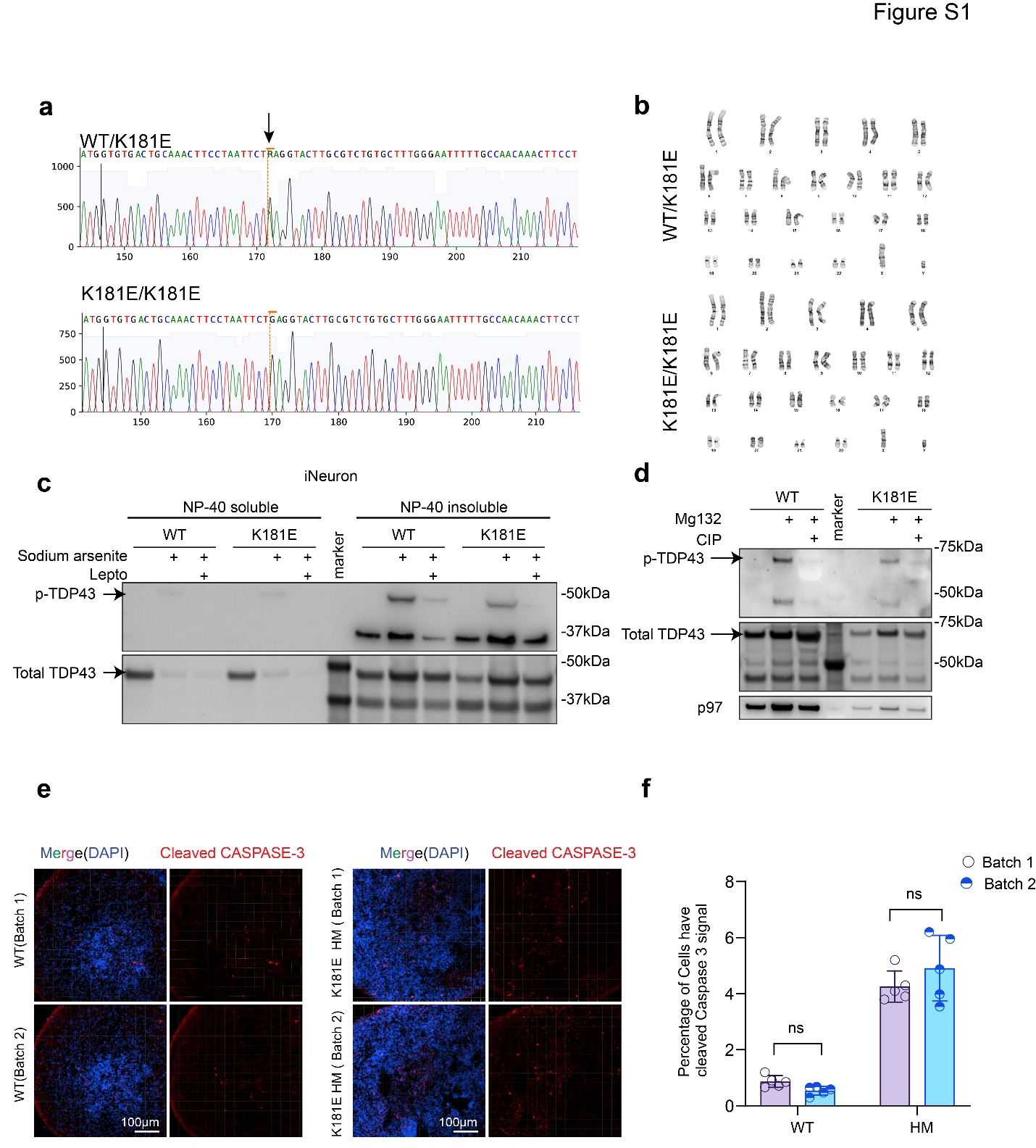
**

**Figure S1 Validation of TDP-43 K181E mutation knock-in and characterization of TDP-43 hyperphosphorylation in iNeuron derived excitatory neuron.**

**(a)** Sanger sequencing confirms iPSC clones with one allele of TDP-43 K181E (top) or homozygous for TDP-43 K181E (bottom).

**(b)** Chromosome analysis confirms the correct karyotype for TDP-43 K181E clones.

(**c**) Hyperphosphorylation of endogenous TDP-43 (arrow) was only detected in NP-40 insoluble fraction of mature i^3^Neurons when oxidative stress was induced by sodium arsenite and was suppressed when cells were co-treated with Leptomycin B (Lepto). i^3^Neurons of the indicated genotypes were treated with DMSO as a control, or with sodium arsenite (10 µM) or sodium arsenite and Leptomycin B (200 nM) at day 15 in differentiation. Cells were harvested at day 17 and lysed in a buffer containing NP40. NP40 insoluble fractions were further treated with an SDS-containing buffer before immunoblotting analysis.

(**d**) The specificity of the phosphor-TDP-43 antibody was tested in HEK293T cells transfected with plasmids expressing ΔNLS-TDP-43-mGreenLantern and ΔNLS-TDP-43 (K181E)-mGreenLantern. Where indicated, cells were treated with the proteasome inhibitor MG132 (10 µM). A fraction of the lysate was treated with a phosphatase (CIP) before immunoblotting.

**(e)** Immunostaining of cleaved Caspase-3 in two individual batches of WT organoids (left) and TDP-43 K181E HM mutant organoids (right). n= 3-5 organoids, 2-3 iPSC clones, two individual batches.

**(f)** Quantification of percentage of cells have cleaved Caspase-3 in e, n= 3-5 organoids, 2-3 iPSC clones, two individual batches. 2way ANOVA. Error bars indicate mean ± s.e.m, ns (no significant).


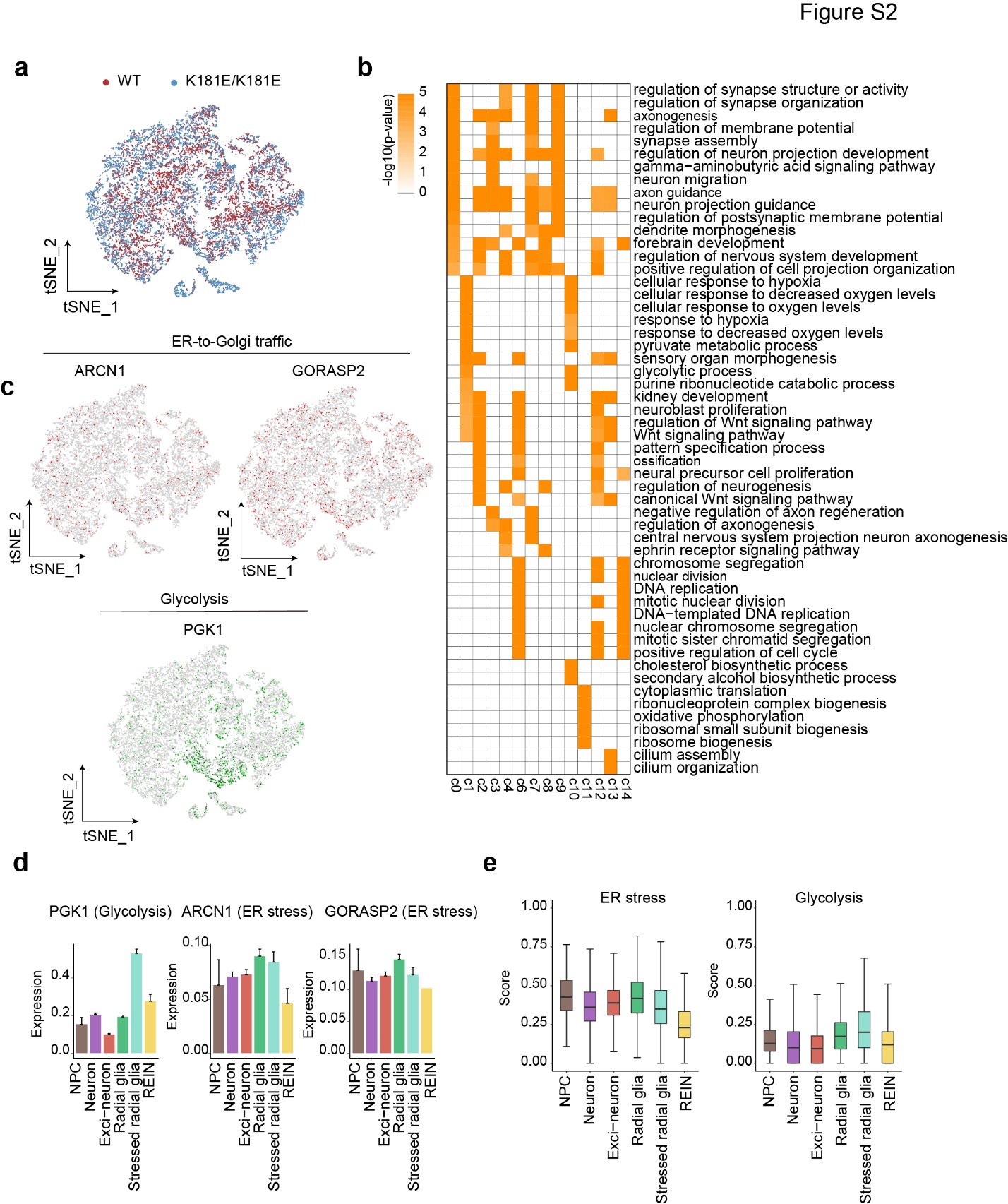


**Figure S2 Single-cell transcriptomic analysis reveals genotype-specific clustering and differential enrichment of ER stress and glycolytic pathways.**

**(a)** T-distributed Stochastic Neighbor Embedding (**t-SNE**) of 16,549 single cells colored by sample genotypes.

**(b)** Heatmap showing the GO enriched pathways in each cell cluster. The significance was determined by adjusted p-value <0.05. Top 5 biological pathways for each cell cluster were displayed based on p-value.

**(c)** Feature plots showing the expression of genes involved in ER-to-Golgi traffic (ARCN1 and GORASP2) and glycolysis (PGK1).

**(d)** Bar graphs showing the relative expression of the indicated genes in each cell type. Shown are mean ± s.e.m.

**(e)** Box plots showing pathway score of ER stress (left) and glycolysis (right). Genes involved in the pathways were obtained from the GSEA database. The pathway scores were calculated as average expression of the genes.

**
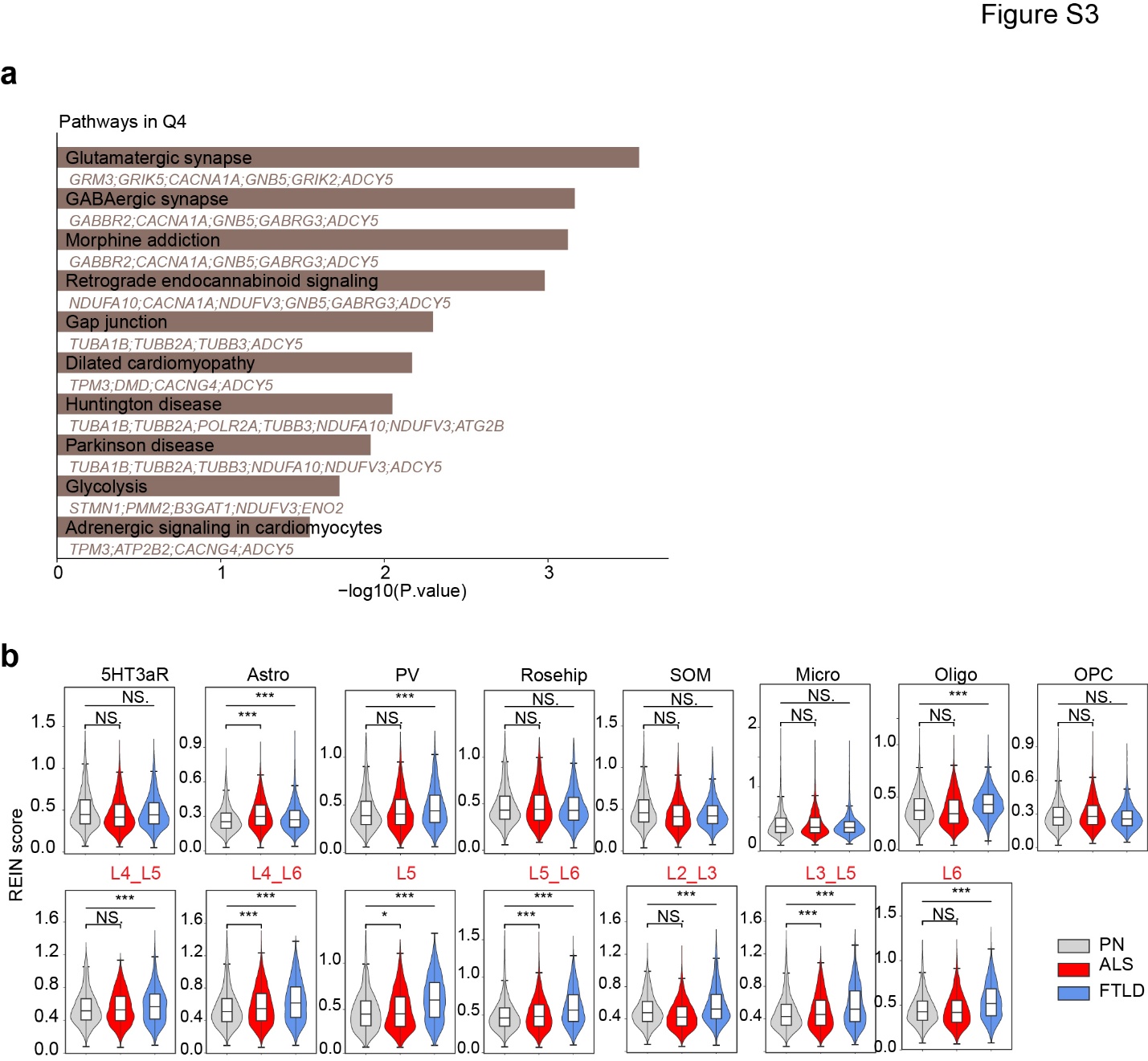
**

**Figure S3 KEGG pathway enrichment for genes in Q4 and cell type specific REIN scores in ALS/FTD patients.**

**(a)** Top enriched KEGG pathways of the genes in Q4 (n=144) from (Fig. 2a).

(**b**) Violin plots of REIN scores in different cell types from ALS/FTD patients (* p < 0.05, *** **p** < 0. 001 by one-tailed unpaired Student’s t-test. NS, non-significant). The REIN score is calculated as the averaged expression of REIN-related genes (Supplementary Table S2). (L4-L6: Cortical layer L4-L6, Micro: microglia, Oligo: oligodendrocyte, OPC: oligodendrocyte progenitor cells, SOM: somatostatin, Rosehip: rosehip neuron, PV: PV neuron, Astro: astrocyte, 5HT3aR: 5HT3aR GABAergic interneuron).

**
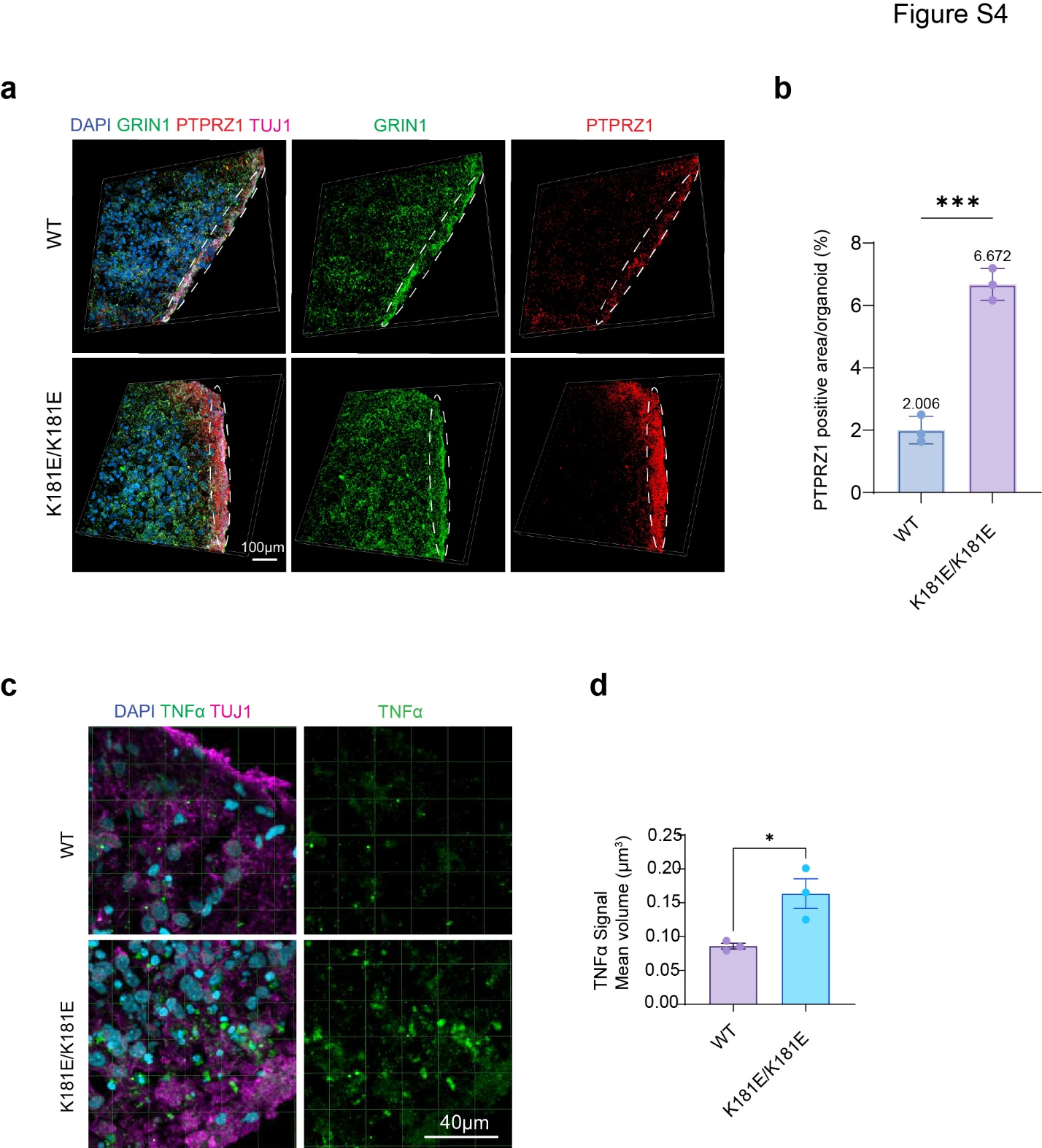
**

**Figure S4 Increased PTPRZ1 and TNF-α expression in TDP-43 K181E Homo mutant organoid.**

**(a)** Representative confocal fluorescence images of immuno-stained organoid sections showing upregulated expression of PTPRZ1(red) in organoids bearing two alleles of K181E (K181E/K181E). Organoids were also stained for neuronal markers TUJ1 (magenta) and GRIN1 (green) as controls. Scale bar, 100 µm. n= 3-5 organoids, 2-3 iPSC clones, two individual batches.

**(b)** Quantification of PTPRZ1 positive area per organoid in 3D fluorescence images of organoids with the indicated genotypes. Error bars indicate mean ± s.e.m., **** p < 0.0001 by unpaired t-test, n=3 organoids.

**(c)** Representative confocal fluorescence images of immuno-stained organoid sections showing upregulated expression of TNF-α (green) in organoids bearing two alleles of K181E (K181E/K181E). Organoids were also stained for neuronal marker TUJ1 (magenta) Scale bar, 100 µm. n= 3-5 organoids, 2-3 iPSC clones, two individual batches.

**(d)** Quantification of TNF-α positive mean spot volume in 3D fluorescence images of organoids with the indicated genotypes. Error bars indicate mean ± s.e.m., **** p < 0.0001 by unpaired t-test, n=3 organoids.


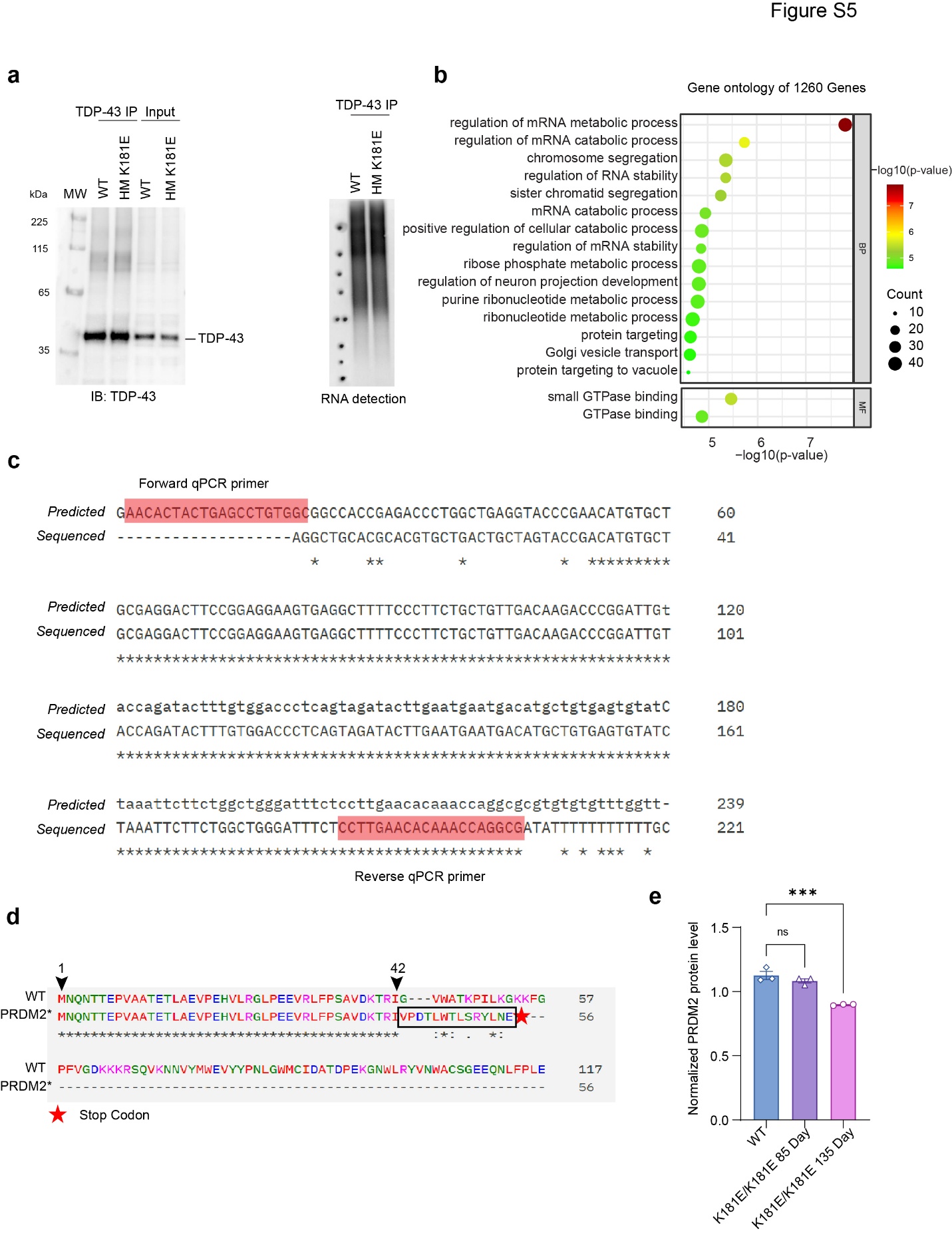


**Figure S5 TDP-43 RNA immunoprecipitation, Gene Ontology analysis of WT-specific bound mRNAs, and validation of PRDM2 cryptic exon inclusion with reduced protein levels in mutant organoids.**

**(a)** Immunoblotting analysis of immunoprecipitated (IP) samples: 10% of IP samples and 1% of input samples were fractionated on a NuPAGE 4-12% Bis-Tris protein gel; Proteins were blotted by anti-TDP43 antibodies (left); RNA in immunoprecipitated samples were visualized by chemiluminescent nucleic acid detection kit (right).

**(b)** Gene Ontology analysis of the mRNAs that only bind WT TDP-43 (Fig. 4b, n=1260) by the eCLIP analysis.

**(c)** DNA sequence alignment reveals the detected CE sequence by Sanger sequencing. Sanger sequencing results of CE fragment amplified by PCR using cDNA as the template. Reference: Predicted CE containing sequence by rMATs. Primers used in qPCR were highlighted in red. Identified CE is indicated by small letters.

**(d)** PRDM2 protein sequence alignment between WT and the translation product of mis-spliced PRDM2 variant (PRDM2*).

**(e)** Normalized PRDM2 protein level in organoids with the indicated genotypes as determined by mass spectrometry. Error bars indicate mean ± s.e.m., one-way ANOVA; **** p < 0.0001, n=3 organoids.

**Supplementary tables:**

Table S1 DEGs for each cluster in scRNA-seq dataset and cell type annotation.

Table S2 Differentially expressed genes in TDP-43 K181E mutant organoids.

Table S3 Gene Ontology analysis of down-regulated TDP-43 binding RNAs identified by eCLIPseq.

Table S4 RNA binding sites detected by eCLIPseq.

Table S5 Detected alternative splicing events in homozygous K181E organoids.

Table S6 Top 20 predicted off target sequences for the sgRNA used in CRISPR editing
